# Poly[μ_5_-(4-meth­oxy­benzene­sulfonato)-sodium]

**DOI:** 10.1107/S1600536813025919

**Published:** 2013-09-25

**Authors:** Suchada Chantrapromma, Nawong Boonnak, Hoong-Kun Fun

**Affiliations:** a Department of Chemistry, Faculty of Science, Prince of Songkla University, Hat-Yai, Songkhla 90112, Thailand; b Faculty of Traditional Thai Medicine, Prince of Songkla University, Hat-Yai, Songkhla 90112, Thailand; c X-ray Crystallography Unit, School of Physics, Universiti Sains Malaysia, 11800 USM, Penang, Malaysia

## Abstract

In the title complex, [Na(C_7_H_7_O_4_S)]_*n*_, the Na^I^ ion is coord­inated in a slightly distorted penta­gonal-bipyramidal environment by seven O atoms [Na—O = 2.3198 (16)–2.5585 (17) Å]. The 4-methoxybenzenesulfonate anions act as bis-chelating and bridging ligands, forming a two-dimensional polymer parallel to (001), which is further linked into a three-dimensional network by weak C—H⋯O hydrogen bonds.

## Related literature

For the appplications of aromatic sulfonic acids, see: Babu *et al.* (2003 ▶); Chanawanno *et al.* (2010 ▶); King (1991 ▶); Ruanwas *et al.* (2010 ▶); Schöngut *et al.* (2011 ▶); Siril *et al.* (2007 ▶); Taylor *et al.* (2006 ▶). For a related structure, see: Smith *et al.* (2004 ▶). For standard bond-lengths, see: Allen *et al.* (1987 ▶).
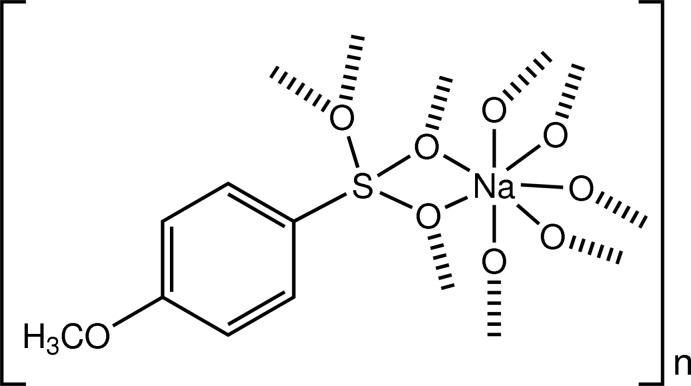


## Experimental

### 

#### Crystal data

[Na(C_7_H_7_O_4_S)]
*M* *_r_* = 210.19
Orthorhombic, 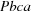

*a* = 8.3121 (8) Å
*b* = 6.0287 (6) Å
*c* = 35.930 (3) Å
*V* = 1800.5 (3) Å^3^
*Z* = 8
Mo *K*α radiation
μ = 0.38 mm^−1^
*T* = 293 K
0.54 × 0.46 × 0.22 mm

#### Data collection

Bruker APEXII CCD area detector diffractometer
Absorption correction: multi-scan (*SADABS*; Bruker, 2009 ▶) *T*_min_ = 0.819, *T*_max_ = 0.920
8793 measured reflections
1769 independent reflections
1720 reflections with *I* > 2σ(*I*)
*R*_int_ = 0.022

#### Refinement

*R*[*F* ^2^ > 2σ(*F* ^2^)] = 0.037
*wR*(*F* ^2^) = 0.101
*S* = 1.20
1769 reflections
119 parameters
H-atom parameters constrained
Δρ_max_ = 0.28 e Å^−3^
Δρ_min_ = −0.28 e Å^−3^

### 

Data collection: *APEX2* (Bruker, 2009 ▶); cell refinement: *SAINT* (Bruker, 2009 ▶); data reduction: *SAINT*; program(s) used to solve structure: *SHELXTL* (Sheldrick, 2008 ▶); program(s) used to refine structure: *SHELXTL*; molecular graphics: *SHELXTL* and *Mercury* (Macrae *et al.*, 2006 ▶); software used to prepare material for publication: *SHELXTL*, *PLATON* (Spek, 2009 ▶), *Mercury* and *publCIF* (Westrip, 2010 ▶).

## Supplementary Material

Crystal structure: contains datablock(s) global, I. DOI: 10.1107/S1600536813025919/lh5648sup1.cif

Structure factors: contains datablock(s) I. DOI: 10.1107/S1600536813025919/lh5648Isup2.hkl

Additional supplementary materials:  crystallographic information; 3D view; checkCIF report

## Figures and Tables

**Table 1:** Hydrogen-bond geometry (Å, °)

*D*—H⋯*A*	*D*—H	H⋯*A*	*D*⋯*A*	*D*—H⋯*A*
C6—H6*A*⋯O1^i^	0.93	2.37	3.282 (3)	165
C7—H7*A*⋯O4^ii^	0.96	2.56	3.407 (4)	148

Symmetry codes: (i) 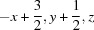; (ii) 
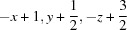
.

**Fig. 1.**
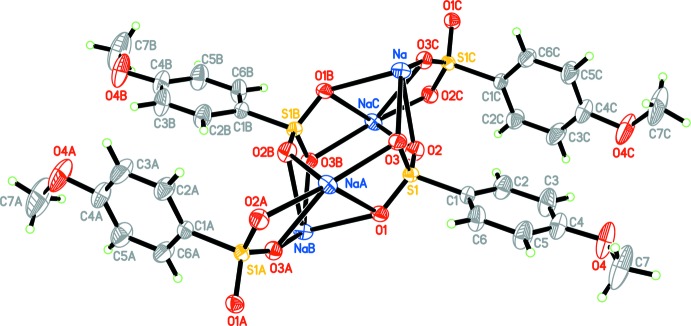
Part of the polymeric structure of (I) showing a tetranuclear cluster with 50% probability displacement ellipsoids. Atoms labelled with the suffix A, B, and C are generated by the symmetry operations (x + 1/2, -y + 1/2, -z + 2), (-x + 1, -y, -z + 2) and (-x + 1/2, y + 1/2, z), respectively.

**Fig. 2.**
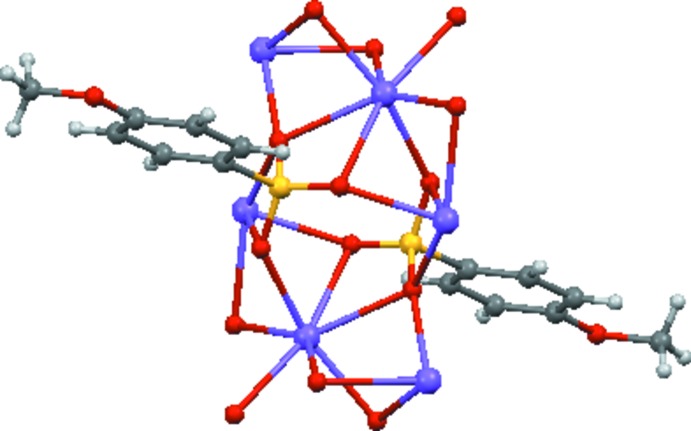
Part of the polymeric structure of (I) showing the coordination environment of NaI ions in (I).

**Fig. 3.**
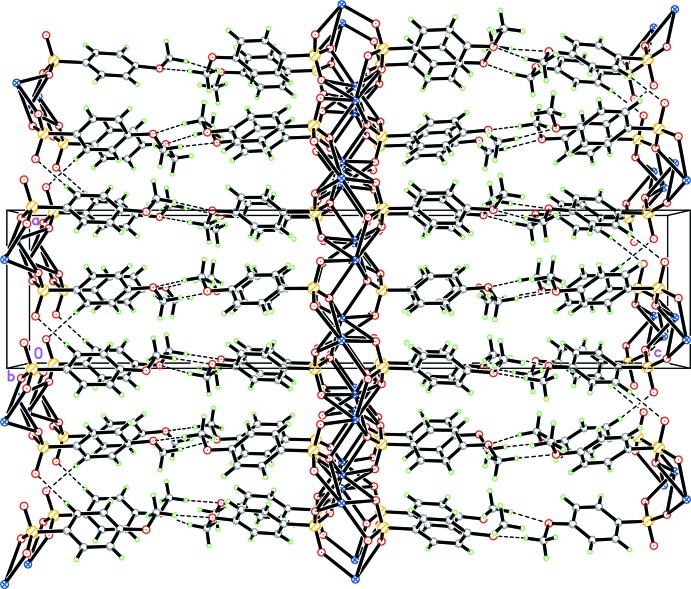
The two-dimensional polymer of (I) viewed along the b axis. Hydrogen bonds are drawn as dashed lines.

**Fig. 4.**
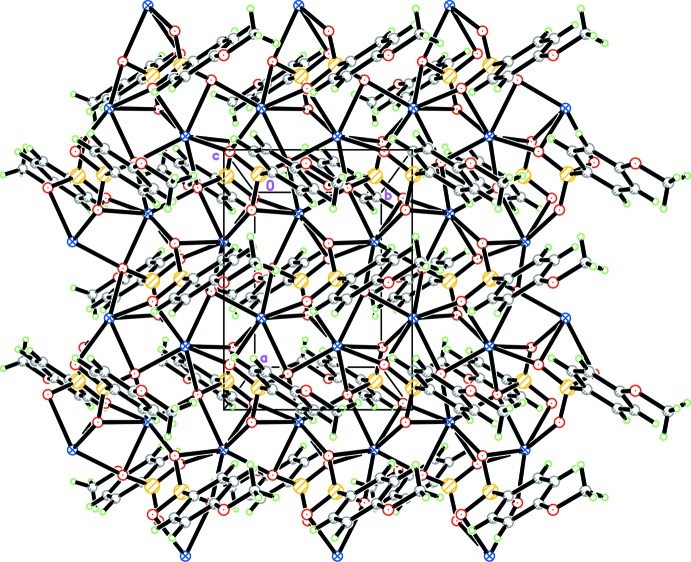
The crystal packing of (I), viewed along the c axis.

## supplementary crystallographic information

### 1. Comment

Aromatic sulfonic acids are one of several useful sulfonic acids which are frequently used as chemical reagents (King, 1991) such as reagents for phenol preparation. They are also widely used as acid catalysts in organic reactions (Siril *et al.*, 2007) such as for the deprotection of *O*-allylphenols (Babu *et al.*, 2003). Salts of benzenesulfonic acid exhibit pharmaceutical and biological activities (Chanawanno *et al.*, 2010; Taylor *et al.*, 2006), and are also used for nonlinear optical material preparations (Ruanwas *et al.*, 2010) and as raw materials in detergent manufacture (Schöngut *et al.*, 2011). Based on these significant roles played by aromatic sulfonic acids, we have synthesized the sodium salt of 4-methoxybenzenesulfonate and herein we report the crystal structure of the title compound (I).

Within the title coordination polymer there are tetranuclear clusters containing four Na^I^ ions and four 4-methoxybenzenesulfonate ligands (Fig. 1). All three O atoms of the 4-methoxybenzenesulfonate ligands are involved in coordination to the Na^I^ ion. The coordination modes of the sulfonate unit are chelating bidentate and bridging monodentate linking two Na^I^ ions. Each Na^I^ is in a distorted pentagonal-bipyramidal geometry (Fig. 2) with three pairs of chelating O atoms from the three bidentate sulfonate groups and one bridging O atom from another monodentate sulfonate group which is also coordinated to a symmetry related Na^I^ ion. The distance of the Na^I^ ion from the mean plane of the O_5_ equatorial atoms is 0.127 Å. Bond lengths (Allen *et al.*, 1987) and angles in the ligand are in normal ranges. The Na—O bond distances in the equatorial plane range from 2.3198 (16) - 2.5585 (17) Å, and the two axial Na—O bond distances are 2.3734 (17) and 2.4637 (16) Å. The O—Na—O bond angles in the equatorial plane are in the range 56.76 (5)–85.88 (6) ° and the axial angle is 158.12 (7)°. These values are comparable to those reported for another Na—O donor complex (Smith *et al.*, 2004). The overall structure is a two-dimensional polymer parallel to (001) (Fig. 3). In addition, weak C—H···O hydrogen bonds (Table 1) link the polymer into a three-dimensional network (Fig. 4).

### 2. Experimental

To a solution of 4-methoxybenzenesulfonyl chloride (3.00 g, 14.50 mmol) in hot methanol, sodium hydroxide (0.58 g, 14.50 mmol) was added. The suspension was stirred for 1 h. The reaction mixture was then cooled to the room temperature and the resulting white solid formed was filtered off and washed with CH_3_OH. Colorless needle-shaped single crystals suitable for X-ray structure determination were recrystallized from a solution of (I) in CH_3_OH by slow evaporation at room temperature over a few days.

### 3. Refinement

All H atoms were fixed geometrically and allowed to ride on their parent atoms, with d(C—H) = 0.93 Å for aromatic and 0.96 for CH_3_. The *U*_iso_ values were constrained to be 1.5*U*_eq_ of the carrier atom for methyl H atoms and 1.2*U*_eq_ for the remaining H atoms. A rotating group model was used for the methyl groups.

### Crystal data

**Table:**

[Na(C_7_H_7_O_4_S)]	*F*(000) = 864
*M**_r_* = 210.19	*D*_x_ = 1.551 Mg m^−^^3^
Orthorhombic, *P**b**c**a*	Mo *K*α radiation, λ = 0.71073 Å
Hall symbol: -P 2ac 2ab	Cell parameters from 1769 reflections
*a* = 8.3121 (8) Å	θ = 2.3–26.0°
*b* = 6.0287 (6) Å	µ = 0.38 mm^−^^1^
*c* = 35.930 (3) Å	*T* = 293 K
*V* = 1800.5 (3) Å^3^	Needle, colorless
*Z* = 8	0.54 × 0.46 × 0.22 mm

### Data collection

**Table:**

Bruker APEXII CCD area detector diffractometer	1769 independent reflections
Radiation source: sealed tube	1720 reflections with *I* > 2σ(*I*)
Graphite monochromator	*R*_int_ = 0.022
φ and ω scans	θ_max_ = 26.0°, θ_min_ = 2.3°
Absorption correction: multi-scan (*SADABS*; Bruker, 2009)	*h* = −8→10
*T*_min_ = 0.819, *T*_max_ = 0.920	*k* = −7→7
8793 measured reflections	*l* = −37→44

### Refinement

**Table:**

Refinement on *F*^2^	Secondary atom site location: difference Fourier map
Least-squares matrix: full	Hydrogen site location: inferred from neighbouring sites
*R*[*F*^2^ > 2σ(*F*^2^)] = 0.037	H-atom parameters constrained
*wR*(*F*^2^) = 0.101	*w* = 1/[σ^2^(*F*_o_^2^) + (0.0372*P*)^2^ + 1.6253*P*] where *P* = (*F*_o_^2^ + 2*F*_c_^2^)/3
*S* = 1.20	(Δ/σ)_max_ = 0.001
1769 reflections	Δρ_max_ = 0.28 e Å^−^^3^
119 parameters	Δρ_min_ = −0.28 e Å^−^^3^
0 restraints	Extinction correction: *SHELXL*, Fc^*^=kFc[1+0.001xFc^2^λ^3^/sin(2θ)]^-1/4^
Primary atom site location: structure-invariant direct methods	Extinction coefficient: 0.042 (3)

### Special details

**Table:**

Geometry. All e.s.d.'s (except the e.s.d. in the dihedral angle between two l.s. planes) are estimated using the full covariance matrix. The cell e.s.d.'s are taken into account individually in the estimation of e.s.d.'s in distances, angles and torsion angles; correlations between e.s.d.'s in cell parameters are only used when they are defined by crystal symmetry. An approximate (isotropic) treatment of cell e.s.d.'s is used for estimating e.s.d.'s involving l.s. planes.
Refinement. Refinement of *F*^2^ against ALL reflections. The weighted *R*-factor *wR* and goodness of fit *S* are based on *F*^2^, conventional *R*-factors *R* are based on *F*, with *F* set to zero for negative *F*^2^. The threshold expression of *F*^2^ > σ(*F*^2^) is used only for calculating *R*-factors(gt) *etc*. and is not relevant to the choice of reflections for refinement. *R*-factors based on *F*^2^ are statistically about twice as large as those based on *F*, and *R*- factors based on ALL data will be even larger.

### Fractional atomic coordinates and isotropic or equivalent isotropic displacement parameters (Å^2^)

**Table:**

	*x*	*y*	*z*	*U*_iso_*/*U*_eq_	
S1	0.50598 (6)	0.11687 (8)	0.950009 (13)	0.0257 (2)	
O1	0.67113 (17)	0.0690 (2)	0.96029 (4)	0.0328 (4)	
O2	0.39805 (19)	−0.0717 (3)	0.95165 (4)	0.0386 (4)	
O3	0.44483 (18)	0.3026 (2)	0.97215 (4)	0.0324 (4)	
O4	0.4881 (4)	0.4270 (4)	0.79459 (6)	0.0843 (8)	
C1	0.5047 (2)	0.2071 (4)	0.90315 (6)	0.0300 (5)	
C2	0.4151 (4)	0.0971 (4)	0.87675 (7)	0.0483 (6)	
H2A	0.3570	−0.0291	0.8831	0.058*	
C3	0.4122 (4)	0.1762 (5)	0.84065 (7)	0.0603 (8)	
H3A	0.3511	0.1036	0.8227	0.072*	
C4	0.4991 (4)	0.3615 (5)	0.83113 (7)	0.0526 (7)	
C5	0.5888 (4)	0.4721 (5)	0.85733 (7)	0.0573 (8)	
H5A	0.6477	0.5974	0.8508	0.069*	
C6	0.5902 (3)	0.3942 (4)	0.89371 (7)	0.0482 (7)	
H6A	0.6493	0.4688	0.9118	0.058*	
C7	0.5762 (7)	0.6163 (7)	0.78319 (10)	0.1151 (19)	
H7A	0.5575	0.6431	0.7572	0.173*	
H7B	0.6888	0.5913	0.7873	0.173*	
H7C	0.5418	0.7428	0.7973	0.173*	
Na	0.18295 (10)	0.12772 (12)	0.98998 (2)	0.0314 (3)	

### Atomic displacement parameters (Å^2^)

**Table:**

	*U*^11^	*U*^22^	*U*^33^	*U*^12^	*U*^13^	*U*^23^
S1	0.0260 (3)	0.0239 (3)	0.0273 (3)	−0.00035 (18)	0.00168 (17)	0.00130 (18)
O1	0.0280 (7)	0.0355 (8)	0.0349 (8)	0.0061 (6)	0.0021 (6)	0.0032 (6)
O2	0.0435 (9)	0.0347 (8)	0.0376 (8)	−0.0131 (7)	0.0013 (7)	0.0018 (6)
O3	0.0333 (8)	0.0311 (8)	0.0330 (8)	0.0078 (6)	0.0023 (6)	−0.0011 (6)
O4	0.143 (2)	0.0803 (16)	0.0299 (10)	−0.0139 (15)	−0.0124 (11)	0.0134 (10)
C1	0.0332 (11)	0.0318 (11)	0.0250 (10)	−0.0001 (8)	−0.0003 (8)	0.0022 (8)
C2	0.0630 (17)	0.0453 (13)	0.0367 (12)	−0.0158 (12)	−0.0037 (11)	−0.0029 (10)
C3	0.086 (2)	0.0616 (17)	0.0337 (13)	−0.0154 (16)	−0.0139 (13)	−0.0076 (12)
C4	0.077 (2)	0.0512 (16)	0.0294 (13)	0.0020 (14)	−0.0036 (11)	0.0044 (11)
C5	0.078 (2)	0.0531 (15)	0.0404 (13)	−0.0231 (15)	−0.0043 (13)	0.0139 (12)
C6	0.0598 (16)	0.0497 (14)	0.0351 (12)	−0.0218 (12)	−0.0102 (11)	0.0076 (10)
C7	0.210 (6)	0.091 (3)	0.0443 (19)	−0.023 (3)	−0.002 (3)	0.0328 (19)
Na	0.0295 (4)	0.0227 (4)	0.0420 (5)	−0.0004 (3)	0.0039 (3)	0.0000 (3)

### Geometric parameters (Å, º)

**Table:**

S1—O2	1.4495 (16)	C3—C4	1.374 (4)
S1—O1	1.4506 (15)	C3—H3A	0.9300
S1—O3	1.4644 (15)	C4—C5	1.374 (4)
S1—C1	1.769 (2)	C5—C6	1.389 (3)
S1—Na^i^	3.0304 (10)	C5—H5A	0.9300
S1—Na	3.0457 (10)	C6—H6A	0.9300
O1—Na^ii^	2.4637 (16)	C7—H7A	0.9600
O1—Na^i^	2.5585 (17)	C7—H7B	0.9600
O2—Na^iii^	2.3734 (17)	C7—H7C	0.9600
O2—Na	2.5572 (18)	Na—O3^iii^	2.3198 (16)
O3—Na^iv^	2.3198 (16)	Na—O2^iv^	2.3734 (17)
O3—Na^i^	2.4384 (17)	Na—O3^v^	2.4384 (17)
O3—Na	2.5021 (17)	Na—O1^ii^	2.4637 (16)
O4—C4	1.374 (3)	Na—O1^v^	2.5585 (17)
O4—C7	1.416 (5)	Na—S1^v^	3.0304 (9)
C1—C6	1.376 (3)	Na—Na^iv^	3.2138 (6)
C1—C2	1.377 (3)	Na—Na^iii^	3.2138 (6)
C2—C3	1.382 (4)	Na—Na^vi^	3.4843 (16)
C2—H2A	0.9300		
			
O2—S1—O1	114.78 (10)	O2^iv^—Na—O3	77.09 (6)
O2—S1—O3	111.27 (9)	O3^v^—Na—O3	140.94 (4)
O1—S1—O3	110.02 (9)	O1^ii^—Na—O3	87.72 (6)
O2—S1—C1	106.03 (10)	O3^iii^—Na—O2	76.94 (6)
O1—S1—C1	108.00 (9)	O2^iv^—Na—O2	104.17 (7)
O3—S1—C1	106.25 (9)	O3^v^—Na—O2	161.64 (6)
O2—S1—Na^i^	132.07 (7)	O1^ii^—Na—O2	79.63 (5)
O1—S1—Na^i^	57.36 (6)	O3—Na—O2	56.76 (5)
O3—S1—Na^i^	52.67 (6)	O3^iii^—Na—O1^v^	141.19 (6)
C1—S1—Na^i^	121.58 (8)	O2^iv^—Na—O1^v^	81.30 (6)
O2—S1—Na	56.77 (7)	O3^v^—Na—O1^v^	57.04 (5)
O1—S1—Na	135.93 (6)	O1^ii^—Na—O1^v^	81.74 (5)
O3—S1—Na	54.65 (6)	O3—Na—O1^v^	84.91 (5)
C1—S1—Na	115.90 (7)	O2—Na—O1^v^	137.65 (6)
Na^i^—S1—Na	94.675 (17)	O3^iii^—Na—S1^v^	113.81 (5)
S1—O1—Na^ii^	138.27 (9)	O2^iv^—Na—S1^v^	83.53 (4)
S1—O1—Na^i^	94.12 (7)	O3^v^—Na—S1^v^	28.52 (4)
Na^ii^—O1—Na^i^	79.55 (5)	O1^ii^—Na—S1^v^	88.14 (4)
S1—O2—Na^iii^	142.94 (10)	O3—Na—S1^v^	112.98 (4)
S1—O2—Na	94.92 (8)	O2—Na—S1^v^	164.05 (5)
Na^iii^—O2—Na	81.26 (5)	O1^v^—Na—S1^v^	28.52 (3)
S1—O3—Na^iv^	162.75 (9)	O3^iii^—Na—S1	104.79 (5)
S1—O3—Na^i^	98.81 (8)	O2^iv^—Na—S1	89.59 (5)
Na^iv^—O3—Na^i^	94.12 (6)	O3^v^—Na—S1	169.25 (5)
S1—O3—Na	96.83 (8)	O1^ii^—Na—S1	84.13 (4)
Na^iv^—O3—Na	83.51 (5)	O3—Na—S1	28.52 (3)
Na^i^—O3—Na	129.48 (6)	O2—Na—S1	28.31 (4)
C4—O4—C7	118.3 (3)	O1^v^—Na—S1	112.25 (4)
C6—C1—C2	120.3 (2)	S1^v^—Na—S1	140.75 (3)
C6—C1—S1	118.91 (17)	O3^iii^—Na—Na^iv^	162.05 (5)
C2—C1—S1	120.73 (18)	O2^iv^—Na—Na^iv^	51.86 (4)
C1—C2—C3	119.3 (2)	O3^v^—Na—Na^iv^	96.89 (4)
C1—C2—H2A	120.3	O1^ii^—Na—Na^iv^	106.30 (5)
C3—C2—H2A	120.3	O3—Na—Na^iv^	45.82 (4)
C4—C3—C2	120.3 (2)	O2—Na—Na^iv^	101.45 (5)
C4—C3—H3A	119.8	O1^v^—Na—Na^iv^	48.93 (4)
C2—C3—H3A	119.8	S1^v^—Na—Na^iv^	72.048 (19)
C3—C4—C5	120.6 (2)	S1—Na—Na^iv^	73.41 (3)
C3—C4—O4	115.9 (3)	O3^iii^—Na—Na^iii^	50.67 (4)
C5—C4—O4	123.4 (3)	O2^iv^—Na—Na^iii^	144.53 (4)
C4—C5—C6	119.0 (2)	O3^v^—Na—Na^iii^	116.30 (4)
C4—C5—H5A	120.5	O1^ii^—Na—Na^iii^	51.52 (4)
C6—C5—H5A	120.5	O3—Na—Na^iii^	95.36 (5)
C1—C6—C5	120.3 (2)	O2—Na—Na^iii^	46.88 (4)
C1—C6—H6A	119.8	O1^v^—Na—Na^iii^	133.13 (4)
C5—C6—H6A	119.8	S1^v^—Na—Na^iii^	130.16 (2)
O4—C7—H7A	109.5	S1—Na—Na^iii^	70.98 (3)
O4—C7—H7B	109.5	Na^iv^—Na—Na^iii^	139.42 (5)
H7A—C7—H7B	109.5	O3^iii^—Na—Na^vi^	44.27 (4)
O4—C7—H7C	109.5	O2^iv^—Na—Na^vi^	102.10 (6)
H7A—C7—H7C	109.5	O3^v^—Na—Na^vi^	41.61 (4)
H7B—C7—H7C	109.5	O1^ii^—Na—Na^vi^	93.85 (5)
O3^iii^—Na—O2^iv^	110.78 (7)	O3—Na—Na^vi^	176.92 (6)
O3^iii^—Na—O3^v^	85.88 (6)	O2—Na—Na^vi^	120.91 (5)
O2^iv^—Na—O3^v^	87.82 (6)	O1^v^—Na—Na^vi^	97.94 (5)
O3^iii^—Na—O1^ii^	91.09 (6)	S1^v^—Na—Na^vi^	69.75 (3)
O2^iv^—Na—O1^ii^	158.12 (7)	S1—Na—Na^vi^	149.03 (4)
O3^v^—Na—O1^ii^	94.46 (6)	Na^iv^—Na—Na^vi^	135.83 (4)
O3^iii^—Na—O3	133.13 (7)	Na^iii^—Na—Na^vi^	83.58 (3)
			
O2—S1—O1—Na^ii^	−46.59 (17)	Na^i^—O3—Na—Na^iv^	90.00 (8)
O3—S1—O1—Na^ii^	79.82 (15)	S1—O3—Na—Na^iii^	30.86 (7)
C1—S1—O1—Na^ii^	−164.62 (13)	Na^iv^—O3—Na—Na^iii^	−166.50 (4)
Na^i^—S1—O1—Na^ii^	78.90 (12)	Na^i^—O3—Na—Na^iii^	−76.51 (7)
Na—S1—O1—Na^ii^	20.4 (2)	S1—O2—Na—O3^iii^	169.52 (8)
O2—S1—O1—Na^i^	−125.50 (8)	Na^iii^—O2—Na—O3^iii^	−47.62 (6)
O3—S1—O1—Na^i^	0.91 (9)	S1—O2—Na—O2^iv^	61.03 (8)
C1—S1—O1—Na^i^	116.48 (9)	Na^iii^—O2—Na—O2^iv^	−156.11 (6)
Na—S1—O1—Na^i^	−58.50 (10)	S1—O2—Na—O3^v^	−169.45 (17)
O1—S1—O2—Na^iii^	48.05 (19)	Na^iii^—O2—Na—O3^v^	−26.6 (2)
O3—S1—O2—Na^iii^	−77.71 (18)	S1—O2—Na—O1^ii^	−96.90 (8)
C1—S1—O2—Na^iii^	167.19 (15)	Na^iii^—O2—Na—O1^ii^	45.95 (5)
Na^i^—S1—O2—Na^iii^	−19.4 (2)	S1—O2—Na—O3	−2.80 (6)
Na—S1—O2—Na^iii^	−82.01 (16)	Na^iii^—O2—Na—O3	140.06 (7)
O1—S1—O2—Na	130.06 (8)	S1—O2—Na—O1^v^	−31.57 (12)
O3—S1—O2—Na	4.30 (10)	Na^iii^—O2—Na—O1^v^	111.29 (8)
C1—S1—O2—Na	−110.81 (8)	S1—O2—Na—S1^v^	−56.4 (2)
Na^i^—S1—O2—Na	62.61 (10)	Na^iii^—O2—Na—S1^v^	86.49 (17)
O2—S1—O3—Na^iv^	−94.5 (3)	Na^iii^—O2—Na—S1	142.86 (10)
O1—S1—O3—Na^iv^	137.1 (3)	S1—O2—Na—Na^iv^	7.82 (8)
C1—S1—O3—Na^iv^	20.5 (4)	Na^iii^—O2—Na—Na^iv^	150.67 (4)
Na^i^—S1—O3—Na^iv^	138.1 (4)	S1—O2—Na—Na^iii^	−142.86 (10)
Na—S1—O3—Na^iv^	−90.1 (3)	S1—O2—Na—Na^vi^	174.81 (6)
O2—S1—O3—Na^i^	127.40 (8)	Na^iii^—O2—Na—Na^vi^	−42.33 (7)
O1—S1—O3—Na^i^	−0.97 (10)	O2—S1—Na—O3^iii^	−10.56 (9)
C1—S1—O3—Na^i^	−117.63 (8)	O1—S1—Na—O3^iii^	−103.07 (11)
Na—S1—O3—Na^i^	131.81 (8)	O3—S1—Na—O3^iii^	174.35 (10)
O2—S1—O3—Na	−4.41 (10)	C1—S1—Na—O3^iii^	82.24 (9)
O1—S1—O3—Na	−132.77 (7)	Na^i^—S1—Na—O3^iii^	−149.16 (5)
C1—S1—O3—Na	110.56 (8)	O2—S1—Na—O2^iv^	−121.97 (9)
Na^i^—S1—O3—Na	−131.81 (8)	O1—S1—Na—O2^iv^	145.51 (10)
O2—S1—C1—C6	176.20 (19)	O3—S1—Na—O2^iv^	62.94 (8)
O1—S1—C1—C6	−60.3 (2)	C1—S1—Na—O2^iv^	−29.17 (9)
O3—S1—C1—C6	57.7 (2)	Na^i^—S1—Na—O2^iv^	99.43 (5)
Na^i^—S1—C1—C6	1.9 (2)	O2—S1—Na—O3^v^	162.0 (3)
Na—S1—C1—C6	115.82 (19)	O1—S1—Na—O3^v^	69.5 (3)
O2—S1—C1—C2	−1.4 (2)	O3—S1—Na—O3^v^	−13.1 (3)
O1—S1—C1—C2	122.1 (2)	C1—S1—Na—O3^v^	−105.2 (3)
O3—S1—C1—C2	−119.9 (2)	Na^i^—S1—Na—O3^v^	23.4 (3)
Na^i^—S1—C1—C2	−175.71 (18)	O2—S1—Na—O1^ii^	79.02 (9)
Na—S1—C1—C2	−61.8 (2)	O1—S1—Na—O1^ii^	−13.49 (13)
C6—C1—C2—C3	0.2 (4)	O3—S1—Na—O1^ii^	−96.07 (8)
S1—C1—C2—C3	177.8 (2)	C1—S1—Na—O1^ii^	171.82 (9)
C1—C2—C3—C4	0.6 (5)	Na^i^—S1—Na—O1^ii^	−59.58 (4)
C2—C3—C4—C5	−0.6 (5)	O2—S1—Na—O3	175.09 (11)
C2—C3—C4—O4	−179.9 (3)	O1—S1—Na—O3	82.58 (12)
C7—O4—C4—C3	−179.6 (4)	C1—S1—Na—O3	−92.11 (11)
C7—O4—C4—C5	1.2 (5)	Na^i^—S1—Na—O3	36.49 (7)
C3—C4—C5—C6	−0.1 (5)	O1—S1—Na—O2	−92.51 (12)
O4—C4—C5—C6	179.1 (3)	O3—S1—Na—O2	−175.09 (11)
C2—C1—C6—C5	−0.9 (4)	C1—S1—Na—O2	92.80 (11)
S1—C1—C6—C5	−178.6 (2)	Na^i^—S1—Na—O2	−138.60 (8)
C4—C5—C6—C1	0.9 (5)	O2—S1—Na—O1^v^	157.60 (9)
S1—O3—Na—O3^iii^	−7.49 (14)	O1—S1—Na—O1^v^	65.09 (12)
Na^iv^—O3—Na—O3^iii^	155.15 (6)	O3—S1—Na—O1^v^	−17.49 (8)
Na^i^—O3—Na—O3^iii^	−114.86 (10)	C1—S1—Na—O1^v^	−109.60 (9)
S1—O3—Na—O2^iv^	−113.99 (8)	Na^i^—S1—Na—O1^v^	19.00 (4)
Na^iv^—O3—Na—O2^iv^	48.64 (6)	O2—S1—Na—S1^v^	158.80 (9)
Na^i^—O3—Na—O2^iv^	138.64 (9)	O1—S1—Na—S1^v^	66.29 (11)
S1—O3—Na—O3^v^	176.15 (9)	O3—S1—Na—S1^v^	−16.29 (8)
Na^iv^—O3—Na—O3^v^	−21.21 (9)	C1—S1—Na—S1^v^	−108.40 (9)
Na^i^—O3—Na—O3^v^	68.79 (11)	Na^i^—S1—Na—S1^v^	20.20 (4)
S1—O3—Na—O1^ii^	81.88 (7)	O2—S1—Na—Na^iv^	−172.01 (8)
Na^iv^—O3—Na—O1^ii^	−115.49 (5)	O1—S1—Na—Na^iv^	95.48 (10)
Na^i^—O3—Na—O1^ii^	−25.49 (8)	O3—S1—Na—Na^iv^	12.91 (7)
S1—O3—Na—O2	2.78 (6)	C1—S1—Na—Na^iv^	−79.21 (8)
Na^iv^—O3—Na—O2	165.42 (7)	Na^i^—S1—Na—Na^iv^	49.40 (2)
Na^i^—O3—Na—O2	−104.59 (9)	O2—S1—Na—Na^iii^	27.79 (8)
S1—O3—Na—O1^v^	163.79 (7)	O1—S1—Na—Na^iii^	−64.73 (10)
Na^iv^—O3—Na—O1^v^	−33.58 (5)	O3—S1—Na—Na^iii^	−147.30 (7)
Na^i^—O3—Na—O1^v^	56.42 (8)	C1—S1—Na—Na^iii^	120.59 (8)
S1—O3—Na—S1^v^	168.89 (5)	Na^i^—S1—Na—Na^iii^	−110.811 (19)
Na^iv^—O3—Na—S1^v^	−28.48 (6)	O2—S1—Na—Na^vi^	−8.67 (11)
Na^i^—O3—Na—S1^v^	61.52 (8)	O1—S1—Na—Na^vi^	−101.19 (12)
Na^iv^—O3—Na—S1	162.63 (10)	O3—S1—Na—Na^vi^	176.24 (12)
Na^i^—O3—Na—S1	−107.37 (11)	C1—S1—Na—Na^vi^	84.13 (12)
S1—O3—Na—Na^iv^	−162.63 (10)	Na^i^—S1—Na—Na^vi^	−147.27 (9)

Symmetry codes: (i) *x*+1/2, −*y*+1/2, −*z*+2; (ii) −*x*+1, −*y*, −*z*+2; (iii) −*x*+1/2, *y*−1/2, *z*; (iv) −*x*+1/2, *y*+1/2, *z*; (v) *x*−1/2, −*y*+1/2, −*z*+2; (vi) −*x*, −*y*, −*z*+2.

### Hydrogen-bond geometry (Å, º)

**Table:**

*D*—H···*A*	*D*—H	H···*A*	*D*···*A*	*D*—H···*A*
C6—H6*A*···O1^vii^	0.93	2.37	3.282 (3)	165
C7—H7*A*···O4^viii^	0.96	2.56	3.407 (4)	148

Symmetry codes: (vii) −*x*+3/2, *y*+1/2, *z*; (viii) −*x*+1, *y*+1/2, −*z*+3/2.
